# Histone demethylase JMJD1A promotes expression of DNA repair factors and radio-resistance of prostate cancer cells

**DOI:** 10.1038/s41419-020-2405-4

**Published:** 2020-04-01

**Authors:** Lingling Fan, Songhui Xu, Fengbo Zhang, Xiaolu Cui, Ladan Fazli, Martin Gleave, David J. Clark, Austin Yang, Arif Hussain, Feyruz Rassool, Jianfei Qi

**Affiliations:** 1 Department of Biochemistry and Molecular Biology, University of Maryland, Baltimore, MD USA; 2 Marlene and Stewart Greenebaum Comprehensive Cancer Center, Baltimore, MD USA; 3 Department of Urology, Beijing Friendship Hospital, Capital Medical University, 100050 Beijing, China; 4 Department of Urology, First Hospital of China Medical University, 110001 Shenyang, China; 5 Vancouver Prostate Centre, University of British Columbia, Vancouver, BC Canada; 6 Department of Anatomy and Neurobiology, University of Maryland, Baltimore, MD USA; 7 Department of Pathology, The Johns Hopkins University, Baltimore, MD USA; 8 Baltimore VA Medical Center, Baltimore, MD USA; 9 Department of Radiation Oncology, University of Maryland, Baltimore, MD USA

**Keywords:** Transcription factors, Prostate cancer

## Abstract

The DNA damage response (DDR) pathway is a promising target for anticancer therapies. The androgen receptor and myeloblastosis transcription factors have been reported to regulate expression of an overlapping set of DDR genes in prostate cancer cells. Here, we found that histone demethylase JMJD1A regulates expression of a different set of DDR genes largely through c-Myc. Inhibition of JMJD1A delayed the resolution of γ-H2AX foci, reduced the formation of foci containing ubiquitin, 53BP1, BRCA1 or Rad51, and inhibited the reporter activity of double-strand break (DSB) repair. Mechanistically, JMJD1A regulated expression of DDR genes by increasing not only the level but also the chromatin recruitment of c-Myc through H3K9 demethylation. Further, we found that ubiquitin ligase HUWE1 induced the K27-/K29-linked noncanonical ubiquitination of JMJD1A at lysine-918. Ablation of the JMJD1A noncanonical ubiquitination lowered DDR gene expression, impaired DSB repair, and sensitized response of prostate cells to irradiation, topoisomerase inhibitors or PARP inhibitors. Thus, development of agents that target JMJD1A or its noncanonical ubiquitination may sensitize the response of prostate cancer to radiotherapy and possibly also genotoxic therapy.

## Introduction

Metastatic castration-resistant prostate cancer (mCRPC) is incurable and underlies the mortality of most prostate cancer (PCa) patients. Factors in the DNA damage repair pathway serve as promising targets for anticancer therapies including CRPC^1–3^.

Methylation of histone 3 lysine-9 (H3K9) is a repressive epigenetic modification. The histone demethylase JMJD1A removes mono- and di-methyl groups from H3K9 to enable transcriptional activation. JMJD1A is reportedly overexpressed and plays a tumor-promoting role in a variety of cancers^4–11^. We previously reported that JMJD1A regulates the activities of androgen receptor (AR) and c-Myc in PCa cells^12,13^. JMJD1A promotes the chromatin recruitment of AR through H3K9 demethylation^13^, and also enhances the generation of AR-V7^14^, a hormone-independent truncated form of AR. The proto-oncogene c-Myc forms a heterodimer with Max, and then binds to the Enhancer Box (E box) sequence to regulate gene expression. We have found that JMJD1A increases AR-dependent transcription of c-Myc in PCa cells; JMJD1A also interacts with E3 ubiquitin ligase HUWE1, an event that blocks HUWE1’s ability to target c-Myc for degradation^12^.

Re-examination of our published profiling array data revealed that knockdown of JMJD1A in PCa cells reduced expression of several key factors that mediate DNA damage responses (DDR). DDR is a coordinated cascade of events that senses DNA damage, signals its presence and mediates its repair. DNA double-strand breaks (DSBs) represent the most deleterious damage and are repaired through nonhomologous end joining (NHEJ) or homologous recombination (HR) pathways. Mutation of several DDR genes (e.g. *BRCA1*, *BRCA2*, *ATM*) is found in a subset of PCa^15^ and confers therapeutic vulnerability^16^. On the other hand, some DDR genes can be transcriptionally upregulated in PCa cells, and confer resistance to genotoxic stress^1–3^. Consistent with a key role for JMJD1A in PCa, we found the significant upregulation of JMJD1A protein in CRPC specimens through a post-transcriptional mechanism (manuscript submitted).

Ubiquitination of proteins regulates numerous cellular processes. After addition of a ubiquitin monomer to a lysine (K) residue(s) of target proteins, additional ubiquitin molecules can be attached through any of eight amine groups in the first molecule—the N terminus (M1), K6, K11, K27, K29, K33, K48, and K63—to form polyubiquitin chains with distinct topologies and functional outcomes^17^. K48-linked polyubiquitination targets proteins for proteasomal degradation, whereas K63-linked polyubiquitination mediates protein−protein interaction. In contrast, little is known about functions of the other “noncanonical” polyubiquitination. HUWE1 reportedly targets substrates for proteasomal degradation via K48-linked polyubiquitination^18,19^. Here, we found that HUWE1 can induce K27- and K29-linked noncanonical polyubiquitination of JMJD1A, an activity that promotes expression of DDR genes through c-Myc.

## Results

### JMJD1A knockdown inhibits expression of DDR genes and resolution of γ-H2AX foci

**Fig. 1:**
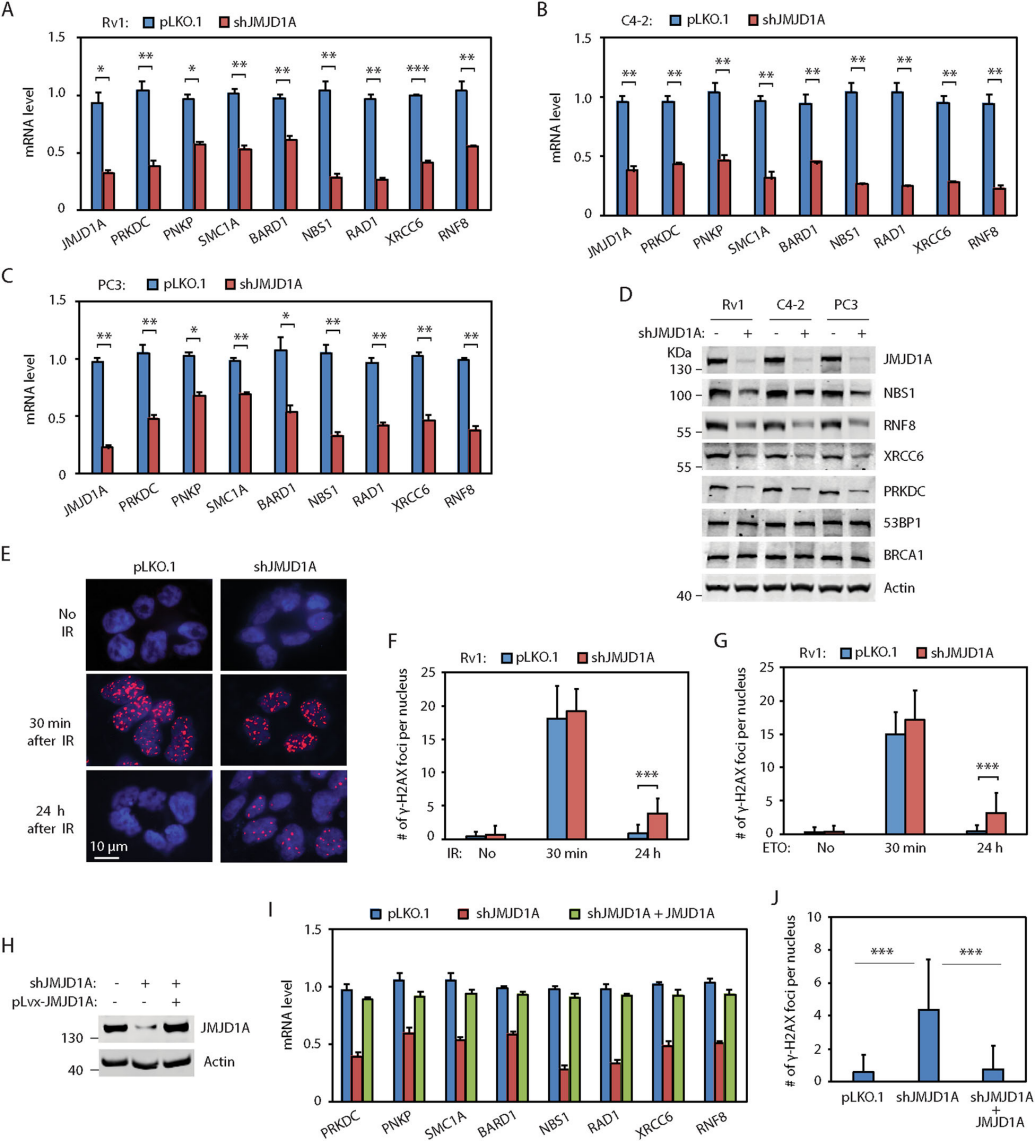
JMJD1A promotes the expression of DDR genes and resolution of γ-H2AX foci. **a, b**, **c** Knockdown of JMJD1A in Rv1 (**a**), C4-2 (**b**), or PC3 (**c**) cells reduced mRNA levels of DDR genes. Cells were transduced with pLKO.1 control or JMJD1A shRNAs for 48h. RNAs were analyzed by qRT-PCR for the indicated genes. **d** Knockdown of JMJD1A in prostate cancer cells reduced the protein level of NBS1, RNF8, PRKDC and XRCC6. **e** Delayed resolution of γ-H2AX foci in the JMJD1A-knockdown Rv1 cells at 24h post IR. Rv1 cells (pLKO.1 control or JMJD1A knockdown) were treated with 2Gy IR, fixed at 30min (middle panel) or 24h (lower panel) post IR, and stained for γ-H2AX. Cells without IR treatment (upper panel) were used as control. Shown are the example staining of γ-H2AX foci. γ-H2AX, red; nuclei, blue. **f** The number of γ-H2AX foci per nucleus was quantified for the cells described in (**e**). Random images of γ-H2AX staining were captured, and the number of γ-H2AX foci per-nucleus was manually counted. Approximately, 50 nuclei were counted for each time point and independent experiments were repeated at least three times. **g** Knockdown of JMJD1A in Rv1 cells delayed the resolution of γ-H2AX foci after ETO treatment. **h** Western blot showing the re-expression of JMJD1A in the JMJD1A-knockdown Rv1 cells. **i** Re-expression of JMJD1A in the JMJD1A-knockdown Rv1 cells restored the expression of DDR gene. **j** Re-expression of JMJD1A in the JMJD1A-knockdown Rv1 cells rescued the resolution of γ-H2AX foci at 24h post IR. The indicated cells were treated with 2Gy IR, and the number of γ-H2AX foci per-nucleus was quantified after 24h. *(*p* < 0.05), **(*p* < 0.01), ***(*p* < 0.001).

To determine the JMJD1A-dependent genes, we previously performed a profiling array study on control and JMJD1A-knockdown Rv1 cells^12,13^. By re-examining the profiling array data (GSE70498), we found that JMJD1A knockdown reduced mRNA levels of several DDR genes (Fig. S1A). Among them, NBS1^20^, RNF8^21^, RAD1^22^, SMC1A^23^ and BARD1^24^ are involved in DSB repair via the HR pathway, while XRCC6^25^, PRKDC^25^ and PNKP^26^ are involved in DSB repair via the NHEJ pathway. We confirmed that knockdown of JMJD1A in PCa cells reduced mRNA levels of these DDR genes (Fig. 1a−c). We also confirmed that JMJD1A knockdown in PCa cells reduced the protein levels of NBS1, RNF8, PRKDC and XRCC6 (Fig. 1d). To assess whether JMJD1A regulates the expression of these DDR genes in vivo, we calculated the JMJD1A score and DDR score in two GEO datasets (GSE21043, GSE35988) of profiling array studies on human PCa tissues. We calculated the JMJD1A score based on the top 100 JMJD1A-activated genes identified in our previous study^12^, to reflect the JMJD1A activity on gene expression. We calculated the DDR score using the eight JMJD1A-dependent DDR genes to reflect their expression levels. We found that the JMJD1A score was positively correlated with the DDR score in metastatic PCa or CRPC, but not in primary PCa (Fig. S1B, C). These results suggest that JMJD1A promotes the expression of DDR genes in metastatic or CRPC tissues, consistent with our findings of elevated JMJD1A staining in CRPC relative to primary PCa tissues.

To determine whether JMJD1A regulates DSB repair, we treated JMJD1A-knockdown Rv1 cells with IR (2 Gy), and performed the γ-H2AX staining (widely used DSB marker) at 30 min or 24 h after IR treatment. At 30 min after IR, we found similar numbers of γ-H2AX foci between control and JMJD1A-knockdown Rv1 cells (Fig. 1e, f). In contrast, at 24 h after IR treatment, the γ-H2AX foci largely disappeared in control cells, whereas some γ-H2AX foci still remained in majority of JMJD1A-knockdown Rv1 cells (Fig. 1e, f). Similarly, knockdown of JMJD1A in C4-2 or PC3 cells delayed resolution of γ-H2AX foci at 24 h post-IR treatment (Fig. S1D, E). We next tested whether JMJD1A knockdown affected the resolution of etoposide (ETO)-induced γ-H2AX foci. At 30 min after ETO treatment (5 μM), we observed similar number of γ-H2AX foci between control and JMJD1A-knockdown PCa cells (Figs. 1g, S1F, G). After 30 min of ETO treatment, we removed ETO from cell culture media and allowed cells to recover for 24 h. At 24 h after ETO removal, the γ-H2AX foci largely disappeared in control cells, whereas some γ-H2AX foci remained in the majority of JMJD1A-knockdown cells (Figs. 1g, S1F, G). To further confirm the specificity of JMJD1A knockdown, we re-expressed the ectopic JMJD1A in the JMJD1A-knockdown Rv1 cells, to the level seen in control cells (Fig. 1h). Of note, the ectopic JMJD1A harbors the silent mutations in the shRNA targeting site and thus escapes the shRNA silencing. Re-expression of JMJD1A in the JMJD1A-knockdown Rv1 cells restored the expression of DDR genes (Fig. 1i) and rescued the resolution of γ-H2AX foci after IR (Fig. 1j).

### JMJD1A knockdown impairs DSB repair

JMJD1A knockdown reduced levels of NBS1 (Fig. 1a−d). NBS1 is a component in the MRE11-RAD50-NBS1 (MRN) complex, which recruits and activates ATM for HR-mediated DSB repair^27,28^. To test whether JMJD1A affects the activation of ATM, we irradiated the JMJD1A-knockdown Rv1 cells (2 Gy) and performed western blotting analysis for phospho-ATM (S1981) and phospho-Chk2 (T68), which are markers of ATM activation. The levels of phospho-ATM and -Chk2 were elevated at 30 min post-IR and reduced to near the basal levels at 24 h post-IR in Rv1 cells (Fig. S2A). Similar patterns of phospho-ATM and -Chk2 were observed between the control and JMJD1A-knockdown cells (Fig. S2A), indicating that JMJD1A does not affect the activation of ATM. We also found that NBS1 knockdown in Rv1 cells did not affect the activation of ATM after ETO treatment (Fig. S2B), suggesting that a small amount of NBS1 may be sufficient for the activation of ATM in Rv1 cells. Thus, JMJD1A-dependent expression of NBS1 in PCa cells does not affect the ATM activation.

**Fig. 2:**
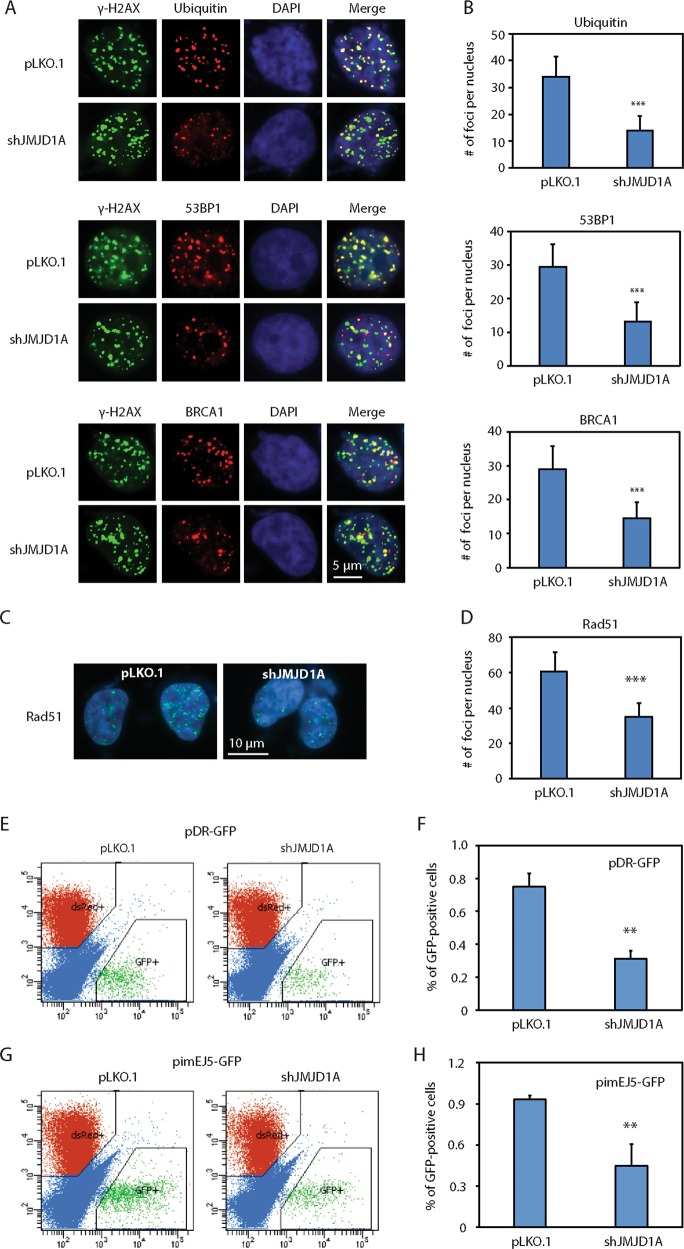
JMJD1A promotes the formation of foci containing ubiquitin, 53BP1 or Rad51, and enhances the reporter activity of DSB repair. **a** Reduced number of DSB foci that are positive for the staining of ubiquitin, 53BP1 or BRCA1 in the JMJD1A-knockdown Rv1 cells. Rv1 cells (pLKO.1 or shJMJD1A) were treated with 5μM of ETO for 30min. Cells were doubly stained for γ-H2AX with ubiquitin (upper panel), 53BP1 (middle panel) or BRCA1 (lower panel). γ-H2AX (green), ubiquitin, 53BP1 or BRCA1 (red), and nuclei (blue). **b** The number of γ-H2AX foci that were positive for ubiquitin, 53BP1 or BRCA1 staining was scored for the cells described in (**a**). The number of foci was quantified as described in Fig. 1f. **c** Reduced number of Rad51 foci in the JMJD1A-knockdown Rv1 cells after DNA damage. Rv1 cells (pLKO.1 control or shJMJD1A) were treated with 5μM of ETO for 1h and stained for Rad51. Shown are example images of staining (Rad51: green; nuclei: blue). **d** The number of Rad51 foci for the cells described in (**c**) was quantified as described in Fig. 1f. **e** Reduced DSB repair by the HR pathway in the JMJD1A-knockdown Rv1 cells. Rv1 cells stably expressing pDR-GFP plasmid were transduced with pLKO.1 or JMJD1A shRNAs for 24h. Cells were then cotransfected with I-SceI and dsRed. dsRed serves as control for transfection efficiency. At 72h post transfection, the GFP-positive cells were measured by flow cytometry. **f** Quantification of GFP-positive cells described in (**e**). **g** Reduced DSB repair by the NHEJ pathway in the JMJD1A-knockdown Rv1 cells. Rv1 cells stably expressing the pimEJ5-GFP plasmid were transduced with pLKO.1 or JMJD1A shRNAs for 24h. The cells were then cotransfected with I-SceI and dsRed. dsRed serves as control for transfection efficiency. At 72h post transfection, the GFP-positive cells were measured by flow cytometry. **h** Quantification of GFP-positive cells described in (**b**). **(*p* < 0.01), ***(*p* < 0.001).

JMJD1A knockdown reduced levels of RNF8 (Fig. 1a−d). RNF8 and RNF168 are ubiquitin ligases that mediate the noncanonical ubiquitination flanking DSB, which leads to recruitment of DNA repair factors such as 53BP1 and RAP80-BRCA1 for HR-mediated DSB repair^21,29^. To determine whether JMJD1A affects the enrichment of ubiquitin, 53BP1 or BRCA1 at the DSB sites, we performed the double staining of γ-H2AX with either ubiquitin, 53BP1 or BRCA1 in the JMJD1A-knockdown Rv1 cells at 30 min after ETO treatment. Although a comparable number of γ-H2AX foci was observed between control and JMJD1A-knockdown Rv1 cells, the number of foci positive for ubiquitin, 53BP1 or BRCA1 was reduced in JMJD1A-knockdown cells (Fig. 2a, b). As a control, knockdown of JMJD1A had no effect on the protein level of 53BP1 or BRCA1 (Fig. 1d). These results suggest that reduced levels of RNF8 in JMJD1A-knockdown cells inhibits ubiquitination and thus recruitment of 53BP1 or BRCA1 at DSB sites.

To further test whether JMJD1A affects the HR-mediated DSB repair, we treated the JMJD1A-knockdown Rv1 cells with ETO for 1 h and then performed the immunostaining for Rad51 foci, a widely used marker for the HR-mediated DSB repair. Compared with control cells, the JMJD1A-knockdown cells showed about 50% reduction in the number of Rad51 foci (Fig. 2c, d). Next, we performed pDR-GFP reporter assays, in which the percentage of GFP-positive cells reflects the HR-mediated repair of DSB induced by I-SceI endonuclease^30^. Compared with control cells, JMJD1A-knockdown Rv1 cells showed about 50% reduction of GFP-positive cells in pDR-GFP reporter assays (Fig. 2e, f). Together, these results indicate that JMJD1A promotes the HR-mediated DSB repair. G1 cell cycle arrest is known to reduce the HR-mediated DSB repair^31^. To determine whether JMJD1A affects cell cycle, we treated the JMJD1A-knockdown Rv1 cells with IR and performed the cell cycle analysis after 24 h. We observed similar levels of IR-induced G1 arrest between control and JMJD1A-knockdown cells (Fig. S2C), indicating the effect of JMJD1A on HR-mediated DSB repair is not due to cell cycle alteration.

JMJD1A knockdown reduced levels of PRKDC and XRCC6 in PCa cells (Fig. 1a−d). PRKDC forms a complex with XRCC6 and XRCC5, and mediates the DSB repair via NHEJ pathway. To test whether JMJD1A affects the DSB repair via NHEJ pathway, we performed pimEJ5-GFP reporter assays, in which the percentage of GFP-positive cells reflects the NHEJ-mediated repair of DSB sites induced by I-SceI endonuclease^32^. Compared with control cells, JMJD1A-knockdown Rv1 cells showed about 50% reduction of GFP-positive cells in pimEJ5-GFP reporter assays (Fig. 2g, h). These results suggest that JMJD1A may also promote the NHEJ-mediated DSB repair.

### JMJD1A promotes DDR gene expression mainly through c-Myc

**Fig. 3:**
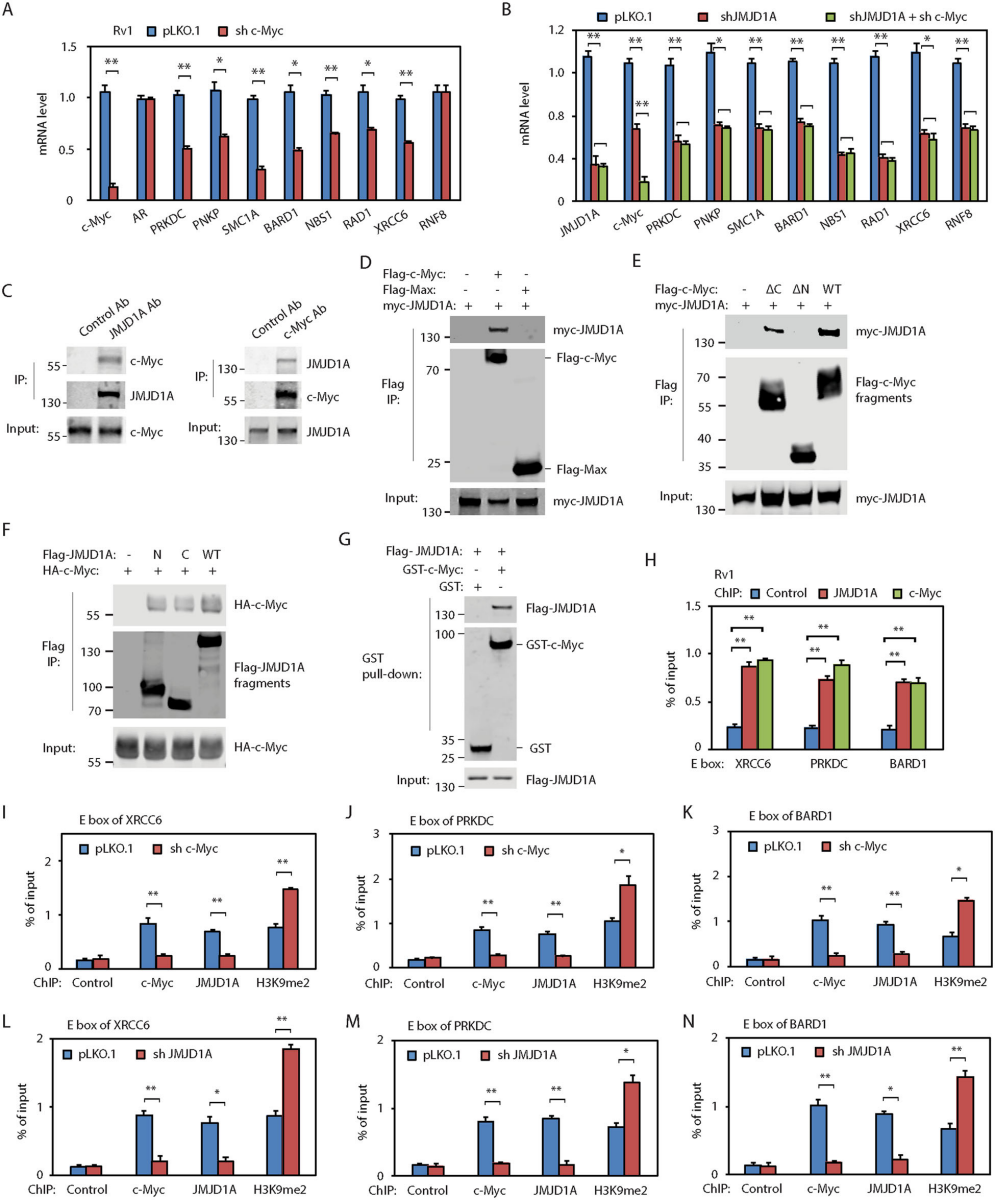
JMJD1A functions as a coactivator for c-Myc for the expression of DDR genes. **a** Knockdown of c-Myc in Rv1 cells reduced mRNA levels of indicated DDR genes. Cells were transduced with pLKO.1 control or c-Myc shRNAs for 48h. RNAs were analyzed by qRT-PCR for the indicated genes. **b** Knockdown of c-Myc in the JMJD1A-knockdown Rv1 cells did not further reduce the expression of DDR genes. Knockdown of JMJD1A alone or knockdown of both JMJD1A and c-Myc was performed on Rv1 cells for 48h. Cells were analyzed by qRT-PCR for the indicated DDR genes. **c** Coimmunoprecipitation between JMJD1A and c-Myc in Rv1 cells. Left panel: JMJD1A was immunoprecipitated from Rv1 cells, and analyzed by western blotting for the coprecipitation of c-Myc. Right panel: c-Myc was immunoprecipitated from Rv1 cells, and analyzed by western blotting for the coprecipitation of JMJD1A. **d** Coprecipitation of myc-JMJD1A with Flag-c-Myc but not with Flag-Max. myc-JMJD1A was coexpressed with Flag-c-Myc or Flag-Max in 293T cells. Cell lysates were subjected to immunoprecipitation (IP) with anti-Flag M2 beads. Bound proteins were analyzed by western blotting with myc or Flag antibodies. **e** Interaction of N-terminal transactivation domain of c-Myc with JMJD1A. myc-JMJD1A was coexpressed with the Flag-tagged truncation mutants of c-Myc and analyzed by Flag IP as described in (**d**). **f** Interaction of N-terminal or C-terminal half of JMJD1A with c-Myc. HA-tagged c-Myc was coexpressed with the Flag-tagged N-terminal half (N) or C-terminal half (C) of JMJD1A, and analyzed by the Flag IP. **g** Direct binding between purified c-Myc and JMJD1A. GST or GST-c-Myc bound on the glutathione agarose was incubated with the recombinant Flag-JMJD1A. The bound proteins were analyzed by western blotting with GST or Flag antibodies. **h** Enrichment of JMJD1A and c-Myc on the E box sites of indicated DDR genes in Rv1 cells. Cells were analyzed by ChIP using antibodies for control IgG, JMJD1A or c-Myc. The precipitated chromatin fragments were analyzed by qPCR with primers targeting the E box region of indicated DDR genes. Data were calculated as the percentage of input. **i**−**k** c-Myc knockdown in Rv1 cells reduced the enrichment of JMJD1A and increased the enrichment of H3K9me2 at the E box region of XRCC6 (**i**), PRKDC (**j**) or BARD1 (**k**) gene. **l**−**n** JMJD1A knockdown in Rv1 cells increased the enrichment of H3K9me2 and decreased the enrichment of c-Myc at the E box region of indicated DDR genes. *(*p* < 0.05), **(*p* < 0.01).

We previously found that JMJD1A regulated AR and c-Myc^12,13^. To determine whether JMJD1A-dependent expression of DDR genes relies on AR or c-Myc, we knocked down AR or c-Myc in PCa cells. Knockdown of c-Myc mRNA (about 90%) in Rv1, C4-2 or PC3 cells partly reduced mRNA levels of JMJD1A-dependent DDR genes except RNF8 (Figs. 3a, S3A, B). In contrast, knockdown of AR in the AR-positive Rv1 or C4-2 cells only reduced mRNA levels of PRKDC but had no effect on the other DDR genes (Fig. S3C, D). To confirm whether JMJD1A regulates the DDR gene expression through c-Myc, we knocked down c-Myc in the JMJD1A-knockdown Rv1 cells (Fig. 3b). Similar mRNA levels of DDR genes were detected between JMJD1A-knockdown and JMJD1A/c-Myc double-knockdown cells (Fig. 3b). Together, these results indicate that JMJD1A-dependent expression of DDR genes is mainly mediated by c-Myc, and JMJD1A may also regulate transcription factor(s) other than AR and c-Myc for expression of RNF8.

### JMJD1A functions as a coactivator for c-Myc

Our previous ChIP-seq study showed that the chromatin fragments bound by JMJD1A were enriched in the E box sequences, which are c-Myc binding motifs^13^. The findings suggest that JMJD1A may also function as a c-Myc co-factor. In support of this possibility, we found that endogenous JMJD1A and c-Myc coimmunoprecipitated in Rv1 cells (Fig. 3c). We also found that ectopic JMJD1A coprecipitated with c-Myc, but not with Max (Fig. 3d). C-Myc has an N-terminal transactivation domain, a central region and a C-terminal DNA-binding domain. To map which domain of c-Myc interacts with JMJD1A, we coexpressed JMJD1A with c-Myc mutants without N-terminal transactivation domain (ΔN) or without C-terminal DNA-binding domain (ΔC) for coimmunoprecipitation experiments. JMJD1A coprecipitated with c-Myc ΔC mutant, but not with ΔN mutant (Fig. 3e), indicating the interaction of JMJD1A with the N-terminal transactivation domain of c-Myc. To determine which region of JMJD1A interacts with c-Myc, we coexpressed c-Myc with N-terminal half or C-terminal half of JMJD1A for coimmunoprecipitation experiments. We found that c-Myc coprecipitated with both N-terminal half and C-terminal half of JMJD1A (Fig. 3f). To determine whether JMJD1A and c-Myc directly interact, we purified the GST-tagged c-Myc (Fig. S3E) and incubated it with the recombinant JMJD1A for an in vitro pull-down experiment. The JMJD1A protein was pulled down by GST-c-Myc, but not by GST protein (Fig. 3g), demonstrating a direct JMJD1A/c-Myc interaction.

To test whether JMJD1A and c-Myc associate with the E box sites of example DDR genes (*XRCC6*, *PRKDC* or *BARD1*), we performed chromatin immunoprecipitation (ChIP) assays using JMJD1A or c-Myc antibodies. We found that both JMJD1A and c-Myc were enriched at the E box sites located in the promoter region of example DDR genes in Rv1 (Fig. 3h) or C4-2 (Fig. S3F) cells. As a control, no enrichment of JMJD1A or c-Myc was found in a respective intronic region of these DDR gene (Fig. S3G, H), which does not harbor the E box sites. These results suggest that JMJD1A may associate with c-Myc on the E box sites of these DDR genes. To directly test this possibility, we knocked down c-Myc in Rv1 cells and then performed ChIP assays using c-Myc or JMJD1A antibodies. Knockdown of c-Myc reduced the enrichment of c-Myc and JMJD1A, while increasing the enrichment of H3K9me2, at the E box sites of example DDR genes (Fig. 3i−k). Thus, these results indicate that c-Myc mediates the association of JMJD1A to the E box sites of these DDR genes. Further, we performed ChIP assays on the JMJD1A-knockdown Rv1 cells. Compared with control, JMJD1A knockdown increased the enrichment of H3K9me2 and decreased the enrichment of c-Myc at the E box sites of these DDR genes (Fig. 3l−n). These results suggest that JMJD1A may function as a coactivator for c-Myc by removing the repressive H3K9 methylation marks.

### HUWE1 induces the noncanonical ubiquitination of JMJD1A

**Fig. 4:**
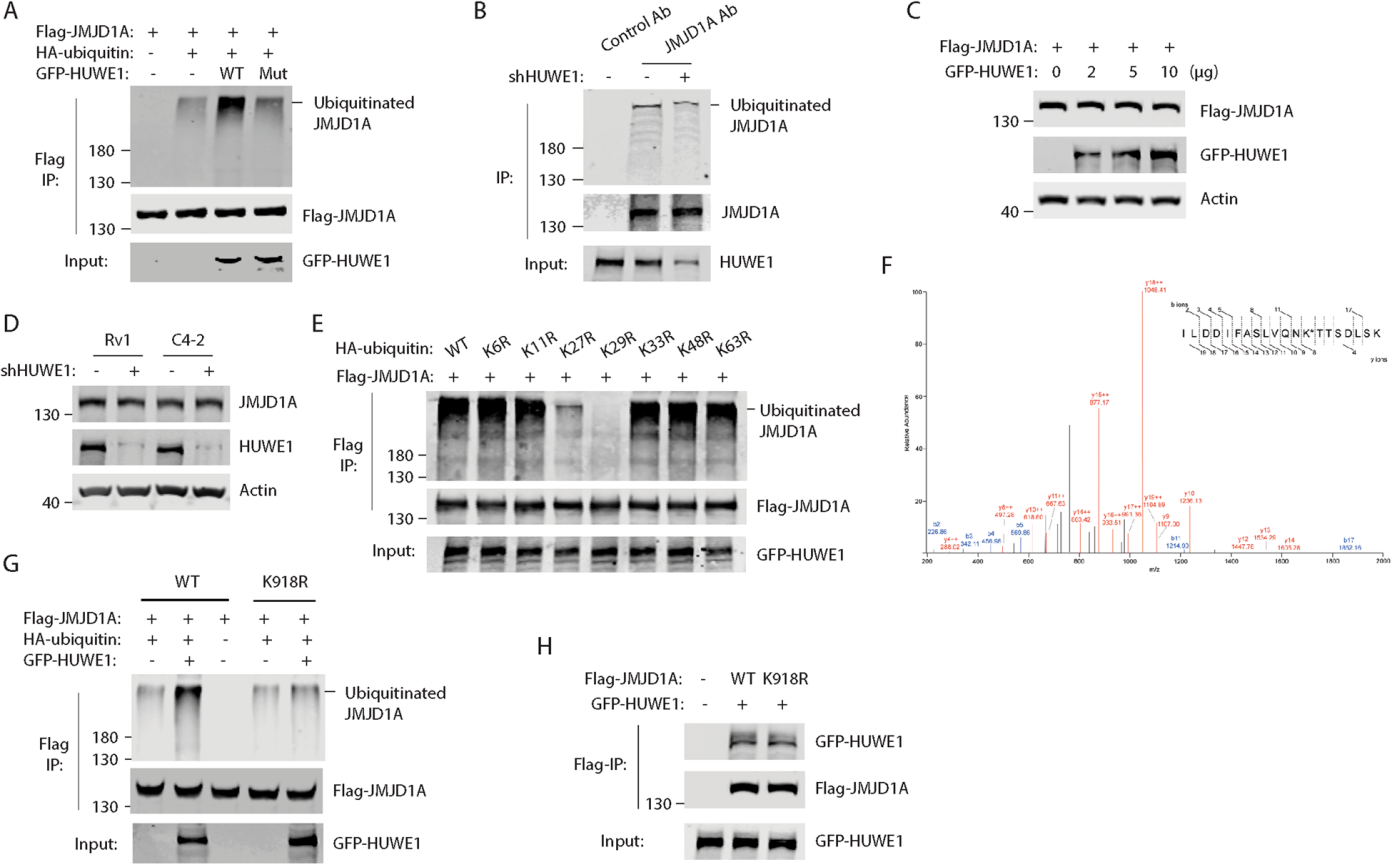
HUWE1 induces the noncanonical ubiquitination of JMJD1A. **a** Polyubiquitination of JMJD1A upon overexpression of HUWE1. **b** Polyubiquitination of JMJD1A by HUWE1 in Rv1 cells. **c** Overexpression of HUWE1 had no effect on the JMJD1A protein level. **d** Knockdown of HUWE1 had no effect on the JMJD1A protein level in Rv1 and C4-2 cells. **e** HUWE1 induced the K27- and K29-linked polyubiquitination of JMJD1A. **f** Mass spectrometry analysis identifies K918 of JMJD1A as the site for HUWE1-induced ubiquitination. Ubiquitinated Flag-JMJD1A was trypsin digested and analyzed by LC-MS/MS. Shown is the MS/MS mass spectrum of the ubiquitinated peptide derived from JMJD1A: ILDDIFASLVQNK*TTSDLSK. a—(sky blue). b—(blue), and y—(red) fragments allow for the mapping of the site of ubiquitination as indicated by asterisk. **g** Validation of JMJD1A K918 as the site for HUWE1-induced ubiquitination. Flag-JMJD1A (WT or K918R) was cotransfected with HA-ubiquitin and GFP-HUWE1 in 293T cells. Flag-JMJD1A was immunoprecipitated and analyzed by western blotting with HA antibodies to detect the JMJD1A ubiquitination. **h** JMJD1A-K918R mutation does not affect its interaction with HUWE1. GFP-HUWE1 was cotransfected with Flag-JMJD1A (WT or K918R) in 293T cells. Flag-JMJD1A was immunoprecipitated and analyzed by western blotting to detect the coprecipitation of GFP-HUWE1.

We previously identified an interaction between JMJD1A and ubiquitin ligase HUWE1^12^. Therefore, we set to determine whether HUWE1 could ubiquitinate JMJD1A by coexpressing Flag-JMJD1A, HA-ubiquitin and GFP-HUWE1 (wild-type or catalytic-inactive) in 293T cells. Flag-JMJD1A was immunoprecipitated and analyzed by western blotting with HA antibodies to detect ubiquitination. We found that overexpression of wild type, but not the catalytic-inactive, GFP-HUWE1 induced polyubiquitination of Flag-JMJD1A (Fig. 4a). To determine whether endogenous JMJD1A was ubiquitinated, we immunoprecipitated JMJD1A from Rv1 cells (control or HUWE1-knockdown) and analyzed by western blotting with ubiquitin antibodies. Polyubiquitination of JMJD1A was detected in control cells, but reduced in HUWE1-knockdown cells (Fig. 4b). Coexpression of Flag-JMJD1A with increasing amounts of GFP-HUWE1 had no effect on levels of Flag-JMJD1A protein in 293T cells (Fig. 4c), and knockdown of HUWE1 in PCa cells had no effect on JMJD1A protein levels (Fig. 4d). As HUWE1-mediated ubiquitination of JMJD1A does not induce JMJD1A degradation, these results suggest the noncanonical ubiquitination of JMJD1A by HUWE1.

To determine the ubiquitin chain topology on JMJD1A, we coexpressed Flag-JMJD1A and GFP-HUWE1 with the various HA-ubiquitin mutants (lysine to arginine). Coexpression of the ubiquitin K27R or K29R mutant significantly reduced JMJD1A ubiquitination, whereas other ubiquitin mutants induced JMJD1A ubiquitination to a similar degree as the wild-type ubiquitin (Fig. 4e). These results suggest that HUWE1 induces the formation of K27- and K29-linked ubiquitin chains on JMJD1A.

To determine the acceptor site(s) of ubiquitin chains on JMJD1A, we induced the JMJD1A ubiquitination by coexpressing Flag-JMJD1A, GFP-HUWE1 and HA-ubiquitin in 293T cells. We purified the ubiquitinated JMJD1A for in-gel trypsin digestion and mass spectrometry analysis to determine ubiquitinated lysine(s). The results identified the amino acid K918 of JMJD1A as the potential ubiquitination site (Fig. 4f). To validate this result, we made the JMJD1A K918R mutant. Coexpression of GFP-HUWE1 and HA-ubiquitin induced the ubiquitination of wild-type JMJD1A, but not the JMJD1A K918R mutant (Fig. 4g). As a control, similar levels of GFP-HUWE1 were coprecipitated with wild-type or K918R mutant JMJD1A (Fig. 4h), indicating that the K918R mutation does not affect the JMJD1A/HUWE1 interaction. Thus, these results confirm that HUWE1 induces ubiquitination of JMJD1A at K918.

### Noncanonical ubiquitination of JMJD1A promotes expression of DDR genes

**Fig. 5:**
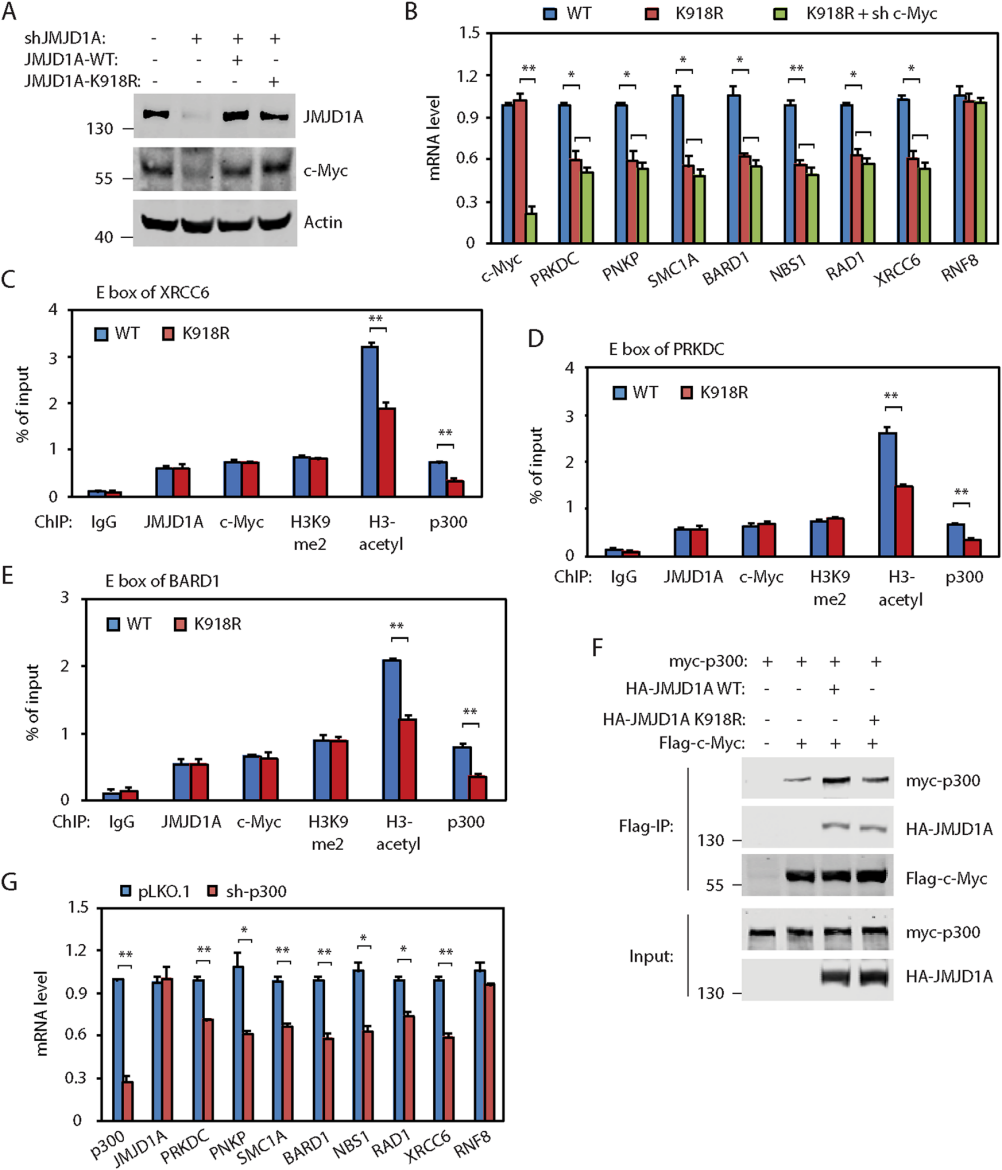
Noncanonical ubiquitination of JMJD1A enhances the recruitment of p300 to c-Myc for the expression of DDR genes. **a** Expression of similar levels of ectopic JMJD1A-WT or JMJD1A-K918R in the JMJD1A-knockdown Rv1 cells. JMJD1A-knockdown Rv1 cells were transduced with the lentiviral constructs of JMJD1A (WT or K918R) to replace the endogenous JMJD1A with the ectopic ones. Cell lysates were analyzed by western blotting with antibodies for JMJD1A or c-Myc. **b** Reduced expression of DDR genes in the JMJD1A^K918R^ cells and no further reduction of DDR genes upon c-Myc knockdown in the JMJD1A^K918R^ cells. Rv1 cells (WT, K918R, K918R with c-Myc knockdown) were analyzed by qRT-PCR for the indicated DDR genes. **c**−**e** Reduced enrichment of p300 and acetylated histone H3 at the E box sites of indicated DDR genes in the JMJD1A^K918R^ relative to JMJD1A^WT^ Rv1 cells. Cells were subjected to ChIP assays using the indicated antibodies. The precipitated chromatin fragments were analyzed by qPCR with primers targeting the E box region of XRCC6 (**c**), PRKDC (**d**) or BARD1 (**e**) genes. **f** Noncanonical ubiquitination of JMJD1A at K918 increased the interaction p300 with c-Myc. Flag-c-Myc, HA-JMJD1A (WT or K918R) and myc-p300 were coexpressed in 293T cells. Flag IP was performed and analyzed by western blotting with Flag, HA or myc antibodies. **g** qRT-PCR analysis showing that knockdown of p300 in Rv1 cells reduced mRNA levels of indicated DDR genes. *(*p* < 0.05), ***(*p* < 0.001).

To determine the role of JMJD1A noncanonical ubiquitination, we depleted the endogenous JMJD1A in Rv1 cells using the JMJD1A shRNA, and then re-expressed similar levels of ectopic JMJD1A (wild type or K918R mutant) using lentiviral transduction (Fig. 5a). We named these cells as JMJD1A^WT^ or JMJD1A^K918R^. JMJD1A^WT^ cells have restored expression of DDR genes to the level seen in control cells (Fig. 1h, i). However, compared with JMJD1A^WT^ cells, JMJD1A^K918R^ cells showed reduced mRNA levels of DDR genes except RNF8 (Fig. 5b), a pattern similar to that seen upon c-Myc knockdown (Fig. 3a). Moreover, knockdown of c-Myc in the JMJD1A^K918R^ cells did not further decrease mRNA levels of DDR genes (Fig. 5b), confirming that noncanonical ubiquitination of JMJD1A regulates DDR gene expression through c-Myc.

We next set to determine mechanisms of JMJD1A noncanonical ubiquitination in regulating the c-Myc activity. Western blot analysis showed similar levels of c-Myc protein between JMJD1A^WT^ and JMJD1A^K918R^ cells (Fig. 5a), indicating that JMJD1A noncanonical ubiquitination does not affect the c-Myc level. ChIP-PCR analysis showed that JMJD1A^WT^ and JMJD1A^K918R^ cells exhibited similar enrichment of c-Myc, JMJD1A and H3K9me2 at the E box sites of example DDR genes (Fig. 5c−e). These results indicate that JMJD1A noncanonical ubiquitination does not affect its H3K9 demethylase activity or the chromatin binding of c-Myc and JMJD1A.

To determine whether JMJD1A noncanonical ubiquitination affects other histone marks, we performed ChIP-PCR analysis on JMJD1A^WT^ and JMJD1A^K918R^ cells using a panel of antibodies specific for various histone modifications, including the acetyl-histone H3, H3K9me1, H3K9me3, H3K4me1, H3K4me2, H3K4me3 and H3K27me3. The only changes we observed were reduced histone acetylation marks on the E box sites of example DDR genes in JMJD1A^K918R^ relative to JMJD1A^WT^ cells (Fig. 5c−e). We did not observe changes in other histone marks examined (not shown). It has been reported that JMJD1A-mediated histone demethylation was followed by histone acetylation^33–35^. P300 is one of the key histone acetyltransferases for c-Myc activity^36,37^. Our ChIP-PCR results showed the reduced enrichment of p300 at the E box sites of example DDR genes in JMJD1A^K918R^ relative to JMJD1A^WT^ cells (Fig. 5c−e), suggesting that JMJD1A noncanonical ubiquitination may promote recruitment of p300 to c-Myc. To further test this possibility, we coexpressed Flag-c-Myc, HA-JMJD1A (WT or K918R) and myc-p300 in 293T cells for the Co-IP experiment. Similar levels of JMJD1A WT and K918R were coprecipitated with c-Myc (Fig. 5f), indicating that noncanonical ubiquitination of JMJD1A does not affect its interaction with c-Myc. Coexpression of JMJD1A WT significantly increased the coprecipitation of p300 with c-Myc (Fig. 5f). In contrast, coexpression of JMJD1A K918R only slightly increased the coprecipitation of p300 with c-Myc (Fig. 5f). Moreover, knockdown of p300 in Rv1 cells reduced mRNA levels of DDR genes (Fig. 5g), an outcome similar to those observed in JMJD1A^K918R^ cells (Fig. 5b). Taken together, these results indicate that noncanonical ubiquitination of JMJD1A promotes recruitment of p300 to c-Myc, to enhance the expression of DDR genes.

### PCa cells expressing the JMJD1A-K918R mutant display defect of DSB repair and increased sensitivity to genotoxic stress

**Fig. 6:**
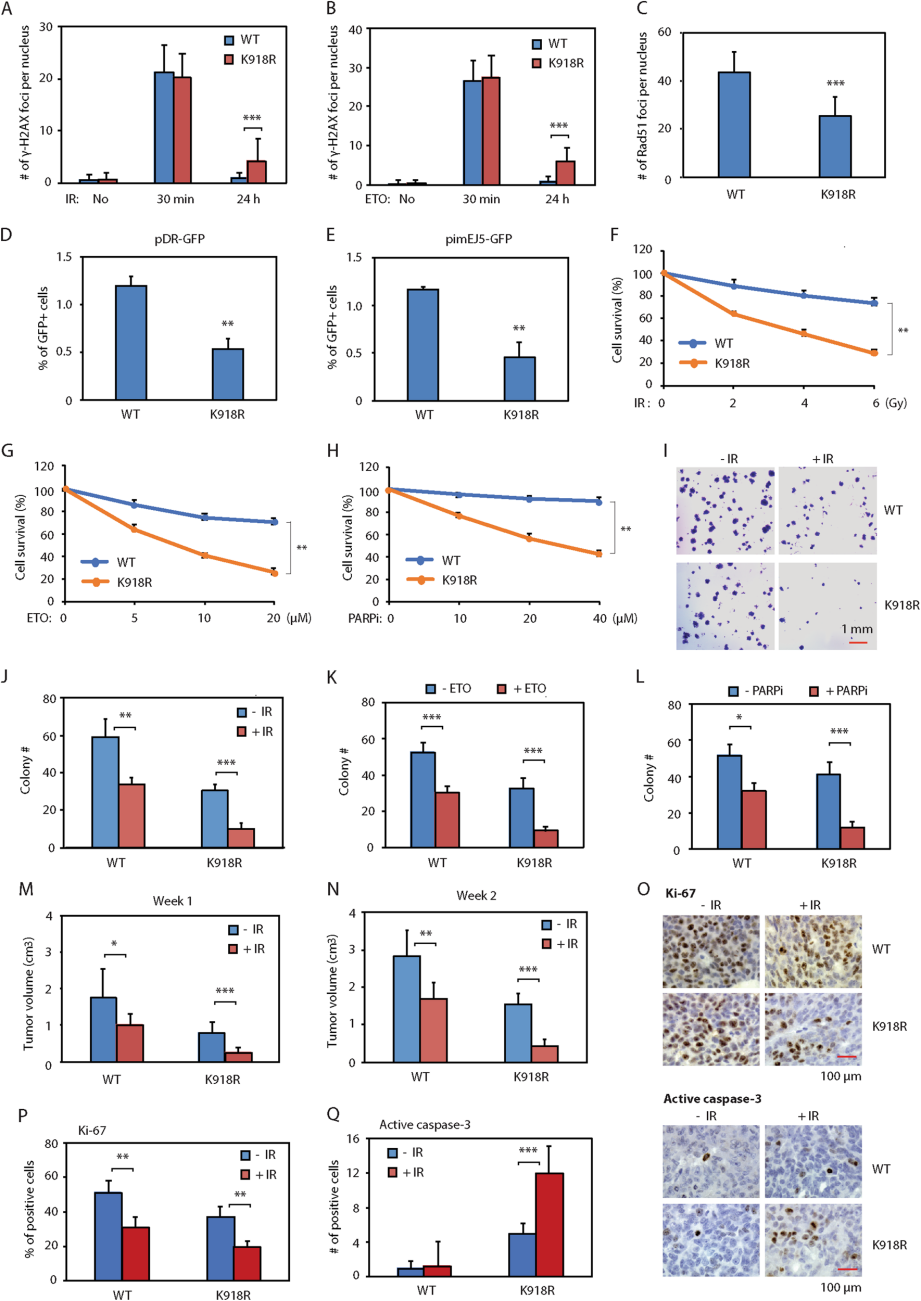
Noncanonical ubiquitination of JMJD1A promotes DSB repair and resistance of prostate cancer cells to genotoxic stress. **a** Delayed resolution of IR-induced γ-H2AX foci in the JMJD1A^K198R^ relative to JMJD1A^WT^ Rv1 cells at 24h post IR. Cells were treated and analyzed as described in Fig. 1f. **b** Delayed resolution of ETO-induced γ-H2AX foci in the JMJD1A^K198R^ relative to JMJD1A^WT^ Rv1 cells. Cells were treated and analyzed as described in Fig. 1g. **c** Reduced Rad51 foci in the JMJD1A^K198R^ relative to JMJD1A^WT^ Rv1 cells after ETO treatment. The procedures are as described in Fig. 2c. **d** Reduced reporter activity of pDR-GFP in the JMJD1A^K198R^ relative to JMJD1A^WT^ Rv1 cells. The procedures are as described in Fig. 2e. **e** Reduced reporter activity of pimEJ5-GFP in the JMJD1A^K198R^ relative to JMJD1A^WT^ Rv1 cells. The procedures are as described in Fig. 2g. **f**−**h** Increased sensitivity of JMJD1A^K918R^ cells to IR (**f**), ETO (**g**) or PARP inhibitor olaparib (**h**) in MTT assays. Rv1 cells (WT or K918R) were treated with the indicated dosage of IR, ETO or olaparib, and analyzed by MTT assays after 48h. The percentage of survival cells was calculated relative to the nontreated cells. **i**−**l** Increased sensitivity of JMJD1A^K918R^ cells to IR (**i**, **j**), ETO (**k**) or olaparib (**l**) in colony formation assays. Rv1 cells (WT or K918R) were treated with IR (2Gy), ETO (2μM) or olaparib (5μM). The number of cell colonies was determined in ten higher-power fields after 3 weeks. **m**, **n** Increased response of JMJD1A^K918R^ xenograft tumors to the IR-induced growth inhibition. The NSG mice harboring xenograft tumors of Rv1 cells (WT or K918R) were treated with 2Gy IR for 2 consecutive days. Xenograft tumor size was measured at 1 (**m**) or 2 (**n**) weeks after IR treatment. **o** Reduced proliferation marker and increased apoptosis marker in the JMJD1A^K918R^ xenograft tumors after IR treatment. IHC staining for Ki-67 or active caspase-3 was performed on the indicated xenograft tumor sections. The staining of Ki-67 (upper panels) or active caspase-3 (lower panel) was visualized with DAB (brown) and counter stained with hematoxylin (blue). **p** Quantification of the percentage of Ki-67-positive nuclei in ten high-power fields described in (**o**). **q** Quantification of the number of active caspase-3-positive nuclei in ten high-power fields described in (**o**). *(*p* < 0.05), **(*p* < 0.01), ***(*p* < 0.001).

JMJD1A^K918R^ cells showed the reduced expression of DDR genes relative to JMJD1A^WT^ cells (Fig. 5b). Consistent with this, relative to JMJD1A^WT^ cells, JMJD1A^K918R^ cells showed the delayed resolution of γ-H2AX foci after IR or ETO treatment (Fig. 6a, b), reduced number of Rad51 foci (Fig. 6c), and decreased reporter activity for DSB repair (Fig. 6d, e). These results indicate that JMJD1A noncanonical ubiquitination promotes DSB repair.

To determine the potential role of JMJD1A noncanonical ubiquitination in the sensitivity of PCa cells to genotoxic stress, we performed MTT assays to evaluate the cell viability in response to IR or ETO treatment. Cancer cells with defect DSB repair are more sensitive to PARP inhibitors^38,39^. To determine the potential role of JMJD1A noncanonical ubiquitination in the sensitivity of PCa cells to PRAR inhibitors, we also performed MTT assays to evaluate the cell viability in response to the PARP inhibitor olaparib. Compared with JMJD1A^WT^ cells, JMJD1A^K918R^ cells exhibited the decreased survival under conditions of IR, ETO or olaparib treatment (Fig. 6f−h). To determine the role of JMJD1A noncanonical ubiquitination in the long-term cell growth/survival, we carried out cell colony formation assays after IR, ETO or olaparib treatment. JMJD1A^WT^ cells showed <50% reduction in colony formation after IR or ETO treatment (Fig. 6i−k). In contrast, JMJD1A^K918R^ cells showed >70% reduction in colony formation after IR or ETO treatment (Fig. 6i−k). Olaparib treatment led to about 20 and 60% reduction in the colony formation by JMJD1A^WT^ and JMJD1A^K918R^ cells respectively (Fig. 6l).

To determine the role of JMJD1A noncanonical ubiquitination in vivo, we injected JMJD1A^WT^ or JMJD1A^K918R^ cells into immuno-deficient mice to form xenograft tumors and then treated the mice with 2 Gy IR for two consecutive days. In the absence of IR treatment, the JMJD1A^K918R^ xenografts showed the reduced tumor growth by about 50% compared to JMJD1A^WT^ xenografts (Fig. 6m, n). After IR treatment, the JMJD1A^WT^ xenografts showed about 40% reduction in the tumor volume, whereas the JMDJ1A^K918R^ xenografts had about 70% reduction (Fig. 6m, n), indicating the increased sensitivity of JMJD1A^K918^ xenografts to the IR-induced growth inhibition. To confirm the results of xenograft studies, we performed the immunohistochemistry staining of a proliferation marker (Ki-67) and an apoptosis marker (active caspase-3) on sections derived from JMJD1A^WT^ and JMJD1A^K918R^ tumor. Compared with JMJD1A^WT^ tumor, JMJD1A^K918R^ tumor showed the reduced staining of proliferation marker with or without IR treatment (Fig. 6o, p), but increased staining of apoptosis marker only after IR treatment (Fig. 6o, q).

Together, the above results indicate that noncanonical ubiquitination of JMJD1A at K918 can promote DDR gene expression, DSB repair, and resistance of prostate cancer cells to IR, topoisomerase inhibitors or PARP inhibitors.

## Discussion

AR and myeloblastosis transcription factors were reported to regulate expression of an overlapping set of DDR genes^1–3^. We found that JMJD1A regulates expression of a different set of DDR genes largely through c-Myc. Our findings are consistent with other studies showing that c-Myc regulates DSB repair in other cell types by promoting expression of DNA repair factors or transduction of DNA damage signaling^40,41^. Of note, although knockdown of AR partly reduced c-Myc mRNA levels (about 40%) in Rv1 and C4-2 cells, it had no effect on mRNA levels of JMJD1A-dependent DDR genes, indicating that the remaining amount of c-Myc in the AR-knockdown cells (about 60%) is sufficient for the expression of these DDR genes. We previously reported that JMJD1A increased transcription and/or stability of c-Myc^12^. Here, we further found that JMJD1A increased the c-Myc chromatin binding through H3K9 demethylation. Thus, JMJD1A is able to regulate the c-Myc activity via multiple mechanisms.

Phosphorylation is known to regulate JMJD1A function^42–44^, while it is unclear if other post-translational modifications modulate the JMJD1A function. We demonstrate that JMJD1A can undergo noncanonical ubiquitination at K918 by HUWE1, and this modification enhances the JMJD1A function with respect to DDR gene expression. HUWE1 is known to either inhibit or increase Myc activity in a context-dependent manner. For example, HUWE1 could inhibit Myc activity by inducing Myc degradation^18,19^. On the other hand, HUWE1 could increase c-Myc activity by degrading the c-Myc inhibitory protein MIZ1^45^ or inducing the K63-linked ubiquitination of c-Myc^46^. Our study now reveals another mechanism for HUWE1 in enhancing c-Myc activity through noncanonical JMJD1A ubiquitination.

HUWE1 induces the formation of K27- and K29-linked ubiquitin chains on JMJD1A. Little is known about the function of noncanonical ubiquitination via K27- or K29-linkage^29,47^. We found that K27- and K29-linked ubiquitination of JMJD1A can enhance recruitment of p300 to some c-Myc targets. Interestingly, others have reported that K63-linked ubiquitination of c-Myc can promote recruitment of p300 to c-Myc targets^46^. Future studies are needed to determine how the noncanonical ubiquitination recruits p300.

Our study identified a role for JMJD1A or its noncanonical ubiquitination in promoting DDR gene expression and DSB repair. Inhibition of JMJD1A function is sufficient to sensitize the response of prostate cancer cells to irradiation, topoisomerase inhibitors or PARP inhibitors. Thus, development of agents that target JMJD1A or its noncanonical ubiquitination may enhance the response of advanced PCa to radiotherapy or genotoxic therapy.

## Materials and methods

### Antibodies and reagents

Antibodies were purchased from the following companies: JMJD1A (12835-1-AP) was from Proteintech (Rosemont, IL). HUWE1 (A300-486A) was from Bethyl Laboratories (Montgomery, TX). RNF8 (sc-271462), BRCA1 (sc-6954), p300 (sc-585), ubiquitin (sc-6085), GFP (sc-8334), HA (sc-7392) and c-Myc (sc-789, sc-40) were from Santa Cruz Biotechnology (Dallas, TX). c-Myc (ab56), PRKDC (ab32566) and Ki67 (ab8191) were from Abcam (Cambridge, UK). NBS1 (3002), p-ATM (4526), ATM (2873), p-Chk2 (2197), Chk2 (2662), XRCC6 (4588), and active caspase-3 (9661) were from Cell Signaling (Danvers, MA). γ-H2AX (05-636), ubiquitin (FK2, ST1200), 53BP1 (MAB3802), Rad51 (ABE257), H3K9me2 (07-441) and acetyl-histone H3 (06-599) were from EMD Millipore (Burlington, MA). Flag (F7425, F3165) and actin (A5441) were from Sigma-Aldrich (St. Louis, MO). Etoposide was from Sigma-Aldrich (St. Louis, MO). Olaparib was from MedChemExpresss (Monmouth Junction, NJ).

### Plasmids

Flag-JMJD1A, myc-JMJD1A, Flag-JMJD1A N-terminal half, Flag-JMJD1A C-terminal half, Flag-c-Myc in the pcDNA3 vector, JMJD1A in the pLvx-IRES-zsGreen1 vector, and GFP-HUWE1 (WT or C4341A mutant) were described previously^12^. C-Myc was cloned into the HA-pcDNA3 vector or pGEX-4T-2 vector. C-Myc ΔN (144-439) or ΔC (1-355) mutant was cloned into the Flag-pcDNA3 vector. HA-JMJD1A or Flag-Max was cloned into pcDNA3 vector. JMJD1A-K918R mutant was generated with the Q5 Site-Directed Mutagenesis Kit (New England Biolabs, Ipswitch, MA). HA-ubiquitin (WT, K6R, K11R, K27R, K29R, K33R, K48R, K63R) in pEF vector were provided by Dr. Ze’ev Ronai (Sanford Burnham Prebys Medical Discovery Institute, La Jolla, CA). JMJD1A, AR, c-Myc, p300, HUWE1 and NBS1 shRNAs were from Sigma-Aldrich (St. Louis, MO). The myc-p300 construct was a gift from Dr. Tso-Pang Yao (Addgene plasmid # 30489). pDR-GFP and pCBASceI were gifts from Dr. Maria Jasin (Addgene plasmid # 26475, # 26477). pimEJ5-GFP was a gift from Dr. Jeremy Stark (Addgene plasmid # 44026).

### Cell lines

Rv1 cells were kindly provided by Dr. James Jacobberger (Case Western Reserve University, Cleveland, Ohio). C4-2 cells were provided by Dr. Leland Chung (Cedars-Sinai Medical Center, Los Angeles, CA). PC3 cells were purchased from American Type Culture Collection (ATCC). Cells were maintained in RPMI 1640 media supplemented with 10% Fetal Bovine Serum (FBS) and antibiotics. All prostate cancer cells were regularly tested to ensure they were mycoplasma-free. The cell lines were periodically authenticated using the STR profiling (Genomics Core Facility, University of Maryland, Baltimore).

### Lentiviral vector packaging and transduction of prostate cancer cells

Lentiviral vectors were packaged in 293T cells by the calcium phosphate transfection. The supernatant containing lentiviral particles were collected 48 h after transfection. Prostate cancer cells were transduced with the supernatant of lentiviral particles in the presence of polybrene (8 µg/ml) for 24 h before replacement with the fresh growth media.

### qRT-PCR analysis

Total RNA was prepared using a total RNA miniprep kit (Sigma-Aldrich, St. Louis, MO) followed by DNase I digestion. cDNA was synthesized using random hexamers. The SYBR Green qPCR analysis was performed with a Mx3005P QPCR system (Agilent Technologies, Santa Clara, CA). Primers for peptidylprolyl isomerase A (PPIA) served as an internal control. Duplicate or triplicate samples were used for qPCR analysis, and independent experiments were repeated at least three times. Primers for qPCR analysis of human gene transcripts were: PPIA: 5′-GACCCAACACAAATGGTTC-3′, 5′-AGTCAGCAATGGTGATCTTC-3′; JMJD1A: 5′-CAGGAGCTCCACATCAGGTT-3′, 5′-TGCATCTTTCACTGCATGGT-3′; c-Myc: 5′-GGCTCCTGGCAAAAGGTCA-3′, 5′-AGTTGTGCTGATGTGTGGAGA-3′; AR: 5′-CTCCGCTGACCTTAAAGACATC-3′; 5′-TGCCCCCTAAGTAATTGTCCTT-3′; PRKDC: 5′-CCTGGCCGGTCATCAACTG-3′, 5′-AGTAAGGTGCGATCTTCTGGC-3′; PNKP: 5′-CTGACCCAGGTTACGGACC-3′, 5′-TCCCGGTAGTTGAGGGGTT-3′; SMC1A: 5′-AACCTGCGGGTAAAGACCCT-3′, 5′-GGCAAAGGTACGGTCCTCAG-3′; BARD1: 5′-CTGCTCGCGTTGTACTAACAT-3′, 5′-TCCAATGCAGTCACTTACACAAT-3′; NBS1: 5′-CACTCACCTTGTCATGGTATCAG-3′, 5′-CTGCTTCTTGGACTCAACTGC-3′; RAD1: 5′-TCTGACCCAACAGATCCAAGA-3′, 5′-TGGCATGTTCTCGGAAATGAA-3′; XRCC6: 5′-GTTGATGCCTCCAAGGCTATG-3′, 5′-CCCCTTAAACTGGTCAAGCTCTA-3′; RNF8: 5′-CCCGGCTTCTTCGTCACAG-3′, 5′-ACCTCGCACCCATCTTCCA-3′. p300: 5′-TTCCCCTAACCTCAATATGGGAG-3′; 5′-GCCTGTGTCATTGGGCTTTTG-3′.

### JMJD1A and DDR score

We downloaded two GEO datasets of profiling arrays studies on human prostate cancer tissues (GSE21043, GSE35988). We used the ssGSEA module (GenePattern) to calculate the JMJD1A or DDR score, similarly to previously described^48^. To evaluate the JMJD1A activity on gene expression, we calculated the JMJD1A score based on the top 100 downregulated genes upon JMJD1A knockdown in Rv1 cells, as revealed in our previous profiling array study^12^. To evaluate the expression of JMJD1A-dependent DDR genes, we calculated the DDR score based on the eight DDR gene identified in this study. The enrichment scores were transformed to a percentile and normalized between 0 and 100. The correlation between JMJD1A score and DDR score in the GEO datasets was evaluated by a scatterplot.

### DSB induction, immunofluorescence staining and quantification of DSB foci

Cells were irradiated with a Co-60 gamma irradiator (2 Gy) or treated with etoposide (ETO, 5 μM) for 30 min to induce DSB. Cells were fixed with 4% paraformaldehyde, incubated with primary antibodies (γ-H2AX, ubiquitin, 53BP1, BRCA1 or Rad51) for 2 h, and followed by incubation with the fluorescein-conjugated secondary antibodies for 1 h at room temperature. After washing, cells were mounted using the Vectashield mounting medium with DAPI. Random images were captured (×63 objective) using a Leica DM4000B fluorescence microscope equipped with cooled CCD imager. The number of foci per-nucleus was manually counted on the images. Approximately, 50 nuclei were counted at each time point and independent experiments were repeated at least three times.

### pDR-GFP and pimEJ5-GFP reporter assay

Rv1 cells were transfected with pDR-GFP or pimEJ5-GFP plasmid using Lipofectamine^TM^ 2000 (Invitrogen, Carlsbad, CA). Cells were selected with puromycin to obtain the stable clones. Rv1 cells that stably expressed pDR-GFP or pimEJ5-GFP were cotransfected with pCBASceI (I-SceI) and dsRed plasmids using Lipofectamine^TM^ 2000. After 72 h, the GFP-positive and dsRed-positive cells were analyzed by a BD LSRII cytometer. dsRed was used as a control for the transfection efficiency. The percentage of GFP-positive cells for pDR-GFP or pimEJ5-GFP reflects the DSB repair via the HR or NHEJ pathway, respectively. The reporter assays were independently performed three times, each time using biological triplicate samples.

### Cell cycle analysis

Cells were fixed with cold 70% ethanol, and incubated with propidium iodine (20 μg/ml) in the presence of RNases (200 μg/ml) for 30 min. Data were acquired on a BD LSRII cytometer. The percentage of cells in each phase of cell cycle (G1, S, or G2) was analyzed by FlowJo software.

### Immunoprecipitation, GST pull-down and western blotting

The procedures of immunoprecipitation and western blotting were detailed in our previous publications^12,14^. GST or GST-c-Myc was purified from BL21 bacteria using glutathione agarose beads (Qiagen). The procedures of GST pull-down were detailed in our previous publication^49^.

### Chromatin immunoprecipitation (ChIP) assay

ChIP assays were performed as detailed in our previous publications^12,14^. The precipitated chromatins were analyzed by qPCR analysis for the E box region of select DDR genes. qPCR analysis for the respective intronic region of select DDR genes was used as negative control. PCR primers used for the E box region: XRCC6: 5′-TACCGGCAACGACTACTGTG-3′, 5′-CTAGCTGGCGAACAACACAA-3′; PRKDC: 5′-AACCAAACAGAGGCAAATGG-3′, 5′-CGCAGGTACTCCGAGCACTA-3′; BARD1: 5′-CACTTAAAGCTGGGTCTGG-3′, 5′-ACCTCCAAAAGGCCAGAGAT-3′. PCR primers used for the intronic region: XRCC6: 5′-TTTAGATCAGCCTGGGCAGT-3′, 5′-ACACCCCAGAAATCCCTCTT-3′; PRKDC: 5′-CCAGCAACAGCATGTCAAGT-3′, 5′-CAAAATAATGGCCCCAAAGA-3′; BARD1: 5′-GCAAAACCCCACCTCTACAA-3′, 5′-GCTGGAGTACAGTGGCATGA-3′.

### Identification of ubiquitination site of JMJD1A by LC-MS analysis

Flag-tagged JMJD1A, HA-ubiquitin and GFP-HUWE1 were cotransfected in 293T cells. Flag-JMJD1A was precipitated, separated with SDS-PAGE and visualized by silver staining. The gel above the Flag-JMJD1A band was cut out, trypsin digested, and analyzed by HPLC-MS/MS. The ubiquitination site was determined by the di-glycine tag (GG, 114.043 Da) on the lysine residue of tryptic peptides derived from JMJD1A.

### MTT assay

Cells were seeded into 96-well plates in triplicate for 24 h. Then, the cells were treated with various dosages of IR or inhibitors (etoposide or olaparib). After 48 h, MTT solution (Sigma) was added to each well, cells were incubated for 3 h and formazan dye was then solubilized with acidic isopropanol. Absorbance at 570 nm was measured using a synergy HT microplate reader (BioTek) and background absorbance at 630 nm was subtracted. The percentage of survival cells was calculated relative to the nontreated cells.

### Cell colony formation assay

Rv1 cells (5 × 10^3^ cells/well) were seeded into six-well plates in triplicate. After 24 h, cells were treated with either IR (2 Gy), ETO (2 μM) or olaparib (5 μM). After 3 weeks, cells were fixed in 4% paraformaldehyde and stained with 0.2% crystal violet. Cell colonies were visualized under a microscope, and the number of colonies (>100 μm in diameter) was determined in ten higher-power fields.

### Xenograft models

NSG mice were purchased from and housed in the animal facility at the University of Maryland School of Medicine. All experiments were approved by the Institutional Animal Care and Use Committee (IACUC #0613011) and conducted following the Institute’s animal policies in accordance with NIH guidelines. Ten mice per group were used for the animal studies based on the published literatures and our previous experience. Eight-week-old male mice were randomly divided into two groups (*n* = 20 per group). JMJD1A^WT^ or JMJD1A^K918R^ Rv1 cells (5 × 10^5^ cells) were subcutaneously (s.c.) injected into the flanks of the mice. After 10 days, half of mice harboring the JMJD1A-WT or JMJD1A-K98R xenografts (*n* = 10) were subjected to total body irradiation (2 Gy) for 2 consecutive days using an XRAD 320 irradiator (Precision X-Ray, North Branford, CT) at the core facility of University of Maryland School of Medicine. The xenograft tumor size was blindly determined at 1 and 2 weeks after irradiation. The tumor volume was calculated using the formula *V* = 1/2(*L* × *W*^2^).

### Immunohistochemistry staining

Sections of Rv1 xenograft tumors were used for immunohistochemistry staining of Ki-67 or active caspase-3. The staining procedures were detailed in our previous publications^12,14^. The images of staining were captured under a Leica DM4000B microscope. For the K-i67 staining, the percentage of positively stained nuclei was calculated in ten high-power fields. For the active caspase-3 staining, the number of positively stained nuclei was counted in ten high-power fields.

### Statistical analysis

In vitro experiments were performed in biological triplicate each time and repeated independently at least three times. Data are presented as the mean ± s.d. Student’s *t* test (two-tailed) was used to compare the difference between two groups of datasets with similar variance. Analysis of variance (ANOVA) test was used to compare the difference of more than two groups of datasets. *p* values less than 0.05 were considered statistically significant. The statistical differences were labeled as *p < 0.05, **p < 0.01, or ***p < 0.001.

## Supplementary information

Supplemental Figure Legend

Supplemental Figure 1

Supplemental Figure 2

Supplemental Figure 3

e-checklist

Author contribution form
