# Supplementary material for: Histone demethylase JMJD1A promotes expression of DNA repair factors and radio-resistance of prostate cancer cells
Source: Cell Death Dis. 2020 Apr 1;11(4):214. doi: 10.1038/s41419-020-2405-4 (PMC7113292; doi:10.1038/s41419-020-2405-4)
Supplement: Supplementary file 1 — Supplemental Figure Legend [file 41419_2020_2405_MOESM1_ESM.docx]

**Supplemental Figure Legends**

**Figure S1. A.** Table showing the fold change of JMJD1A and indicated DDR genes in the JMJD1A-knockdown Rv1 cells relative to the pLKO.1 control, as revealed by our previously published profiling array data (GSE70498). Briefly, Rv1 cells were transduced with lentiviral pLKO.1 or JMJD1A shRNA for 48 hours. Biological duplicate RNA samples (pLKO.1 or JMJD1A-knockdown) were analyzed by the Illumina profiling array (Human-HT12 v4 Expression BeadChip). For the differentially expressed genes (DEGs) between the two groups, an adjusted p-values below 0.05 were considered significant. Here, the differentially expressed DDR genes and their fold changes upon JMJD1A knockdown are shown. **B** and **C.** Correlation between JMJD1A activity and the expression of 8 JMJD1A-dependent DDR genes in metastatic or CRPC specimens. The indicated profiling array datasets of human PCa specimens were downloaded from the GEO database. The JMJD1A score or DDR score was calculated and normalized to a percentile between 0 and 100. The correlation between JMJD1A score and DDR score was examined by a scatterplot. **D** and **E.** Knockdown of JMJD1A in C4-2 (D) or PC3 (E) cells delayed the resolution of γ-H2AX foci at 24 hours post IR. The procedure is as described in Figure 1F. **F** and **G.** Knockdown of JMJD1A in C4-2 (F) or PC3 (G) cells delayed the resolution of γ-H2AX foci after ETO treatment. The procedure is as described in Figure 1G.

**Figure S2. A.** Knockdown of JMJD1A in Rv1 cells had no effect on IR-induced activation of ATM. Rv1 cells (pLKO.1 control or JMJD1A knockdown) were treated with 2 Gy IR. Cell lysates were collected at 30 minutes or 24 hours post IR and analyzed by western blotting for the indicated proteins. **B.** Knockdown of NBS1 in Rv1 cells had no effect on ETO-induced activation of ATM. Rv1 cells (control or NBS1 knockdown) were treated with 5 μM of ETO for 0.5 or 1 hour. Lysates were analyzed by western blotting for the indicated proteins. **C.** Knockdown of JMJD1A in Rv1 cells had little effect on the IR-induced G1 arrest. Cells (control or JMJD1A knockdown) were treated with 2Gy IR. After 24 hours, cells were stained with propidium iodine and analyzed by flow cytometry to determine cell cycle distribution.

**Figure S3.** **A** and **B.** Knockdown of c-Myc in C4-2 (A) or PC3 (B) cells reduced mRNA levels of indicated DDR genes. **C** and **D.** Knockdown of AR in Rv1 (C) or C4-2 (D) cells had no effect on mRNA levels of indicated DDR genes. **E.** Coomassie Blue staining of purified GST and GST-c-Myc protein in the polyacrylamide gel. **F.** ChIP assays showing the enrichment of JMJD1A and c-Myc on the E box sites of indicated DDR genes in C4-2 cells. **G** and **H.** ChIP assays showing no enrichment of JMJD1A or c-Myc on the intronic region of indicated DDR genes in Rv1 (G) or C4-2 (H) cells.
