# Supplementary figures and images for: Histone demethylase JMJD1A promotes expression of DNA repair factors and radio-resistance of prostate cancer cells
Source: Cell Death Dis. 2020 Apr 1;11(4):214. doi: 10.1038/s41419-020-2405-4 (PMC7113292; doi:10.1038/s41419-020-2405-4)

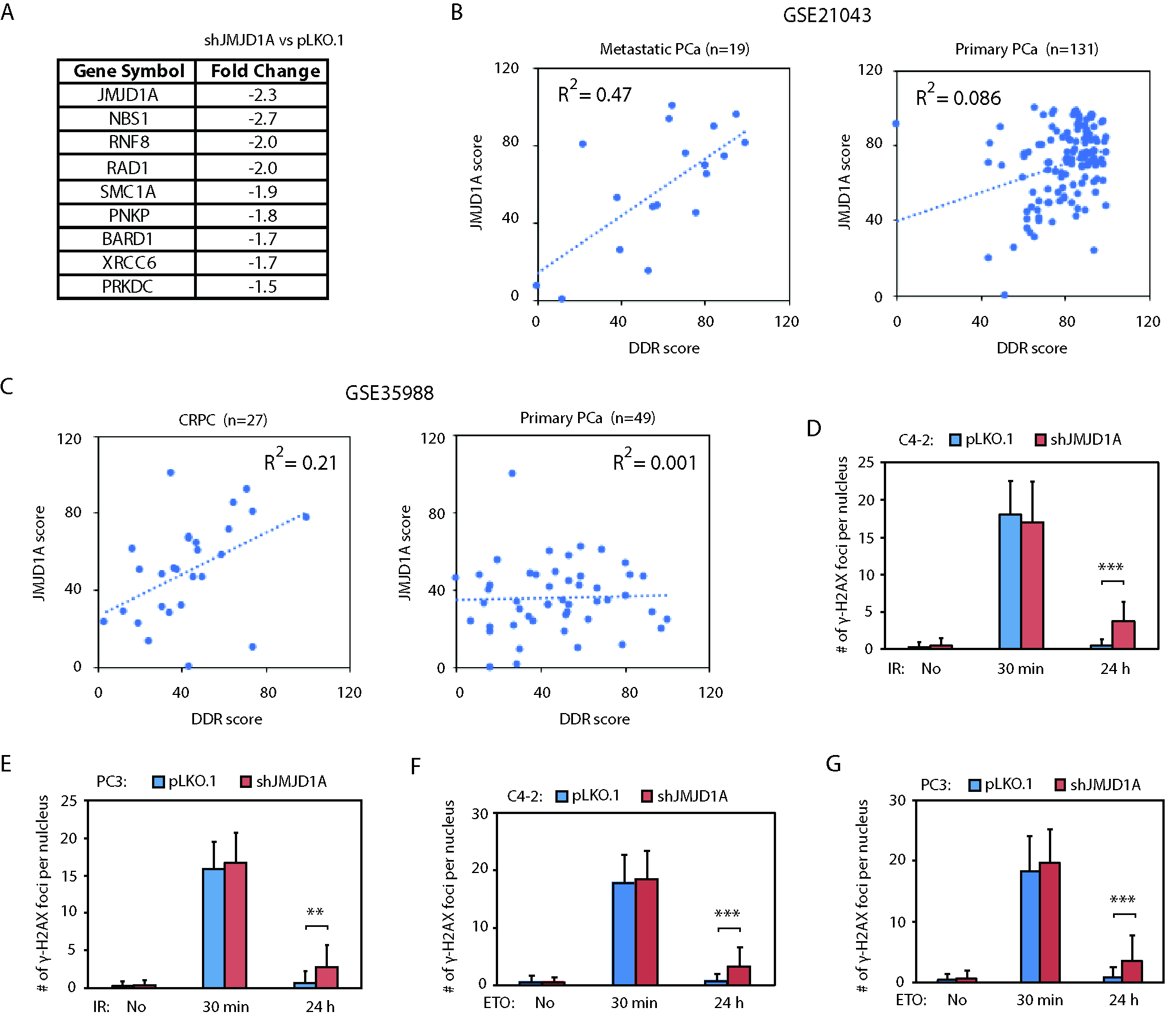

Supplement: Supplementary file 2 — Supplemental Figure 1 [file 41419_2020_2405_MOESM2_ESM.tif]

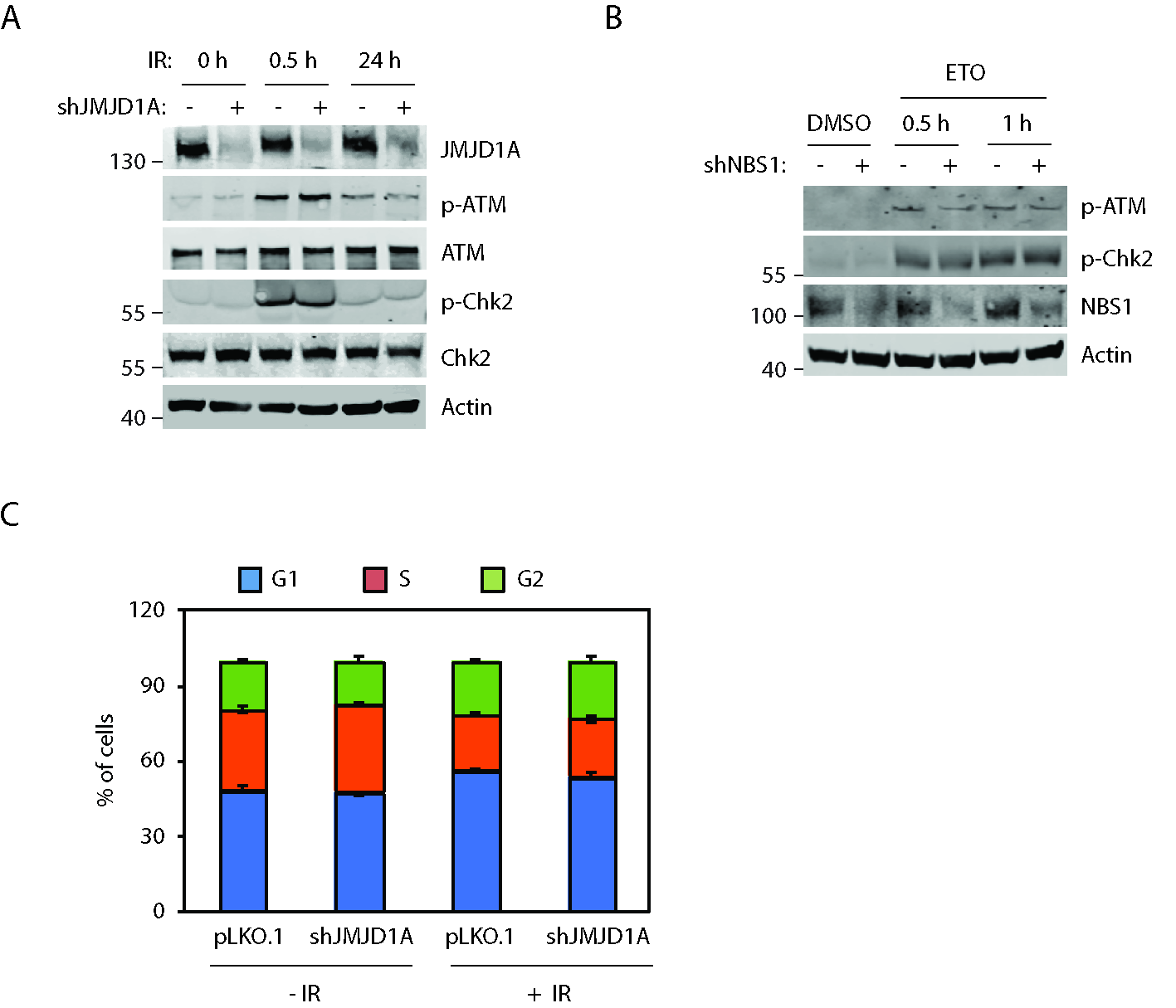

Supplement: Supplementary file 3 — Supplemental Figure 2 [file 41419_2020_2405_MOESM3_ESM.tif]

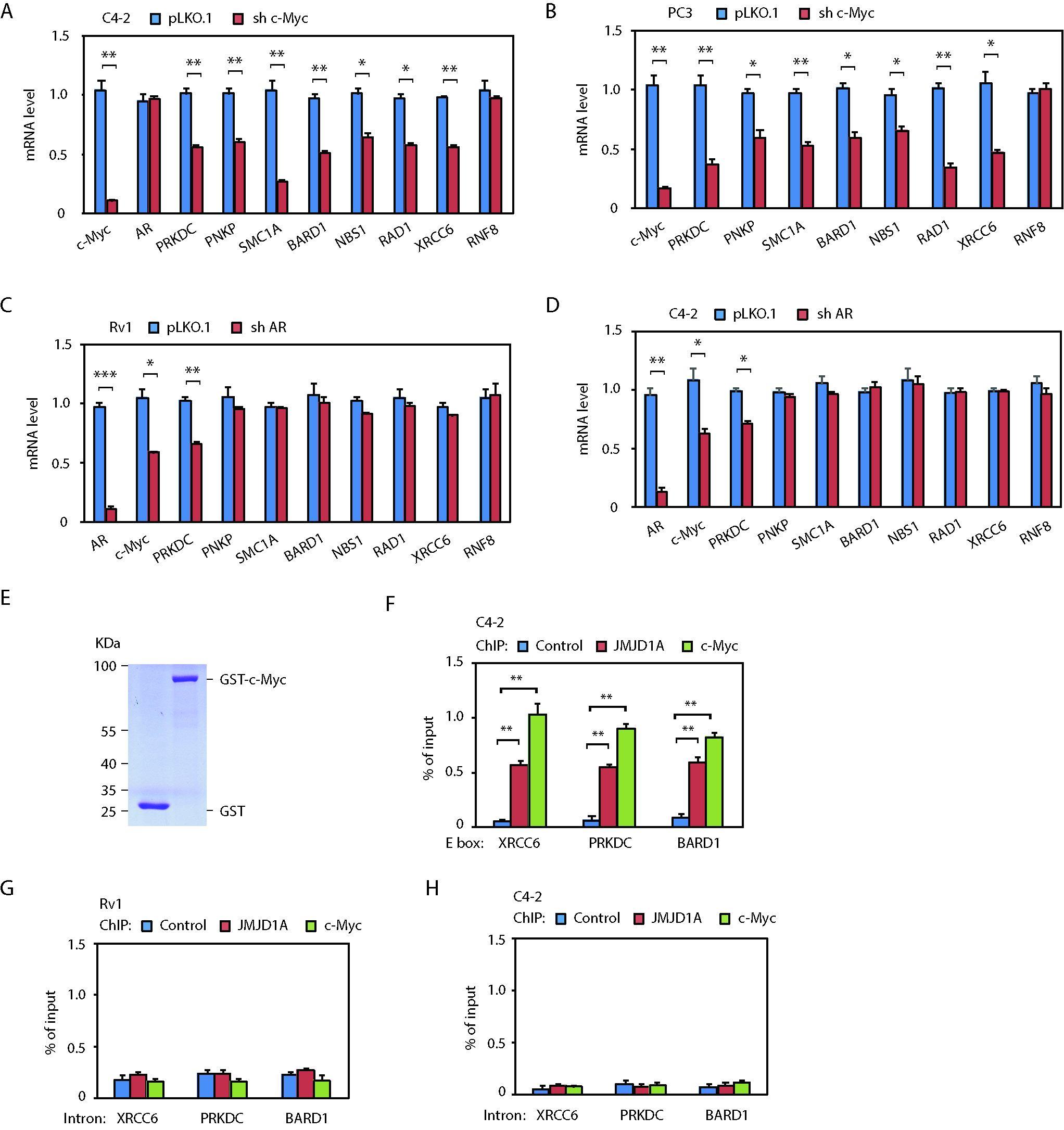

Supplement: Supplementary file 4 — Supplemental Figure 3 [file 41419_2020_2405_MOESM4_ESM.tif]
