# Supplementary material for: Histone demethylase JMJD1A promotes expression of DNA repair factors and radio-resistance of prostate cancer cells
Source: Cell Death Dis. 2020 Apr 1;11(4):214. doi: 10.1038/s41419-020-2405-4 (PMC7113292; doi:10.1038/s41419-020-2405-4)
Supplement: Supplementary file 6 — Author contribution form [file 41419_2020_2405_MOESM6_ESM.pdf]

DECLARATION OF CONTRIBUTIONS TO ARTICLE

ADMC

Manuscript Number: CDDIS-20-0066

Journal Name: *Cell Death & Disease* (the 'Journal')

Proposed Title of the Contribution: Histone demethylase JMJD1A promotes expression DNA repair factors and radio-resistance of prostate cancer cells (the 'Contribution)

Author(s): (the 'Authors')

For all *CDDis* articles, each person named as an author in the published version must be able to show he or she has contributed substantially to the article.

Authorship credit should be based on 1) substantial contributions to conception and design, acquisition of data, or analysis and interpretation of data; 2) drafting the article or revising it critically for important intellectual content; and 3) final approval of the version to be published. Authors should meet conditions 1, 2 and 3.

Any person who cannot be shown to have made a substantial contribution to the article cannot be listed as an author in the final version. The name of any person who is deemed to have made a minor contribution can, however, appear in the Acknowledgments section of the article.

Please complete the table below to indicate the contributions of all named authors to the manuscript.

Author Full Name:	Specification of Contribution to the Manuscript:
Lingling Fan	designed and conducted experiments, analyzed results
Songhui Xu	designed and conducted experiments, analyzed results
Fengbo Zhang	designed and conducted experiments
Xiaolu Cui	designed and conducted experiments
Ladan Fazli	provided resources, advised on data analysis
Martin Gleave	provided resources, advised on data analysis
David J Clark	designed and conducted experiments, analyzed results
Austin Yang	designed experiments, analyzed results
Ari Hussain	provided resources, edit manuscript
Feyruz Rassool	provided resources, designed experiments
Jianfei Qi	Designed experiments, analyzed results, wrote manuscript, supervised all the procedures of experiments

Please complete the table below to indicate the contributions of all named authors to the figures.

Figure 1:

A, B, C: LF
D: SX
E, F, G: LF
H: SX
I, J: LF

Figure 2:

A to H: LF

Figure 3:

A, B: LF
C: SX
D, E, F: LF
G: SX
H to N: LF

Figure 4:

A to E: LF
F: DJC, AY
G: LF
H: SX

Figure 5:

A: SX
B: LF
C, D, E: LF
F: SX
G: LF

Figure 6:

A to E: LF
F, G, H: SX
I, J, K, L: SX
M, N: LF, FZ, XC
O, P, Q: SX

Signed for and on behalf of the Author(s):	Print Name:	Date:
[signature]	Jianfei Qi	02-18-2020
